# Microevolution of the Chromosomal Region of Acute Disease Antigen A (*adaA*) in the Query (Q) Fever Agent *Coxiella burnetii*

**DOI:** 10.1371/journal.pone.0053440

**Published:** 2013-01-03

**Authors:** Dimitrios Frangoulidis, Wolf D. Splettstoesser, Olfert Landt, Jasmin Dehnhardt, Klaus Henning, Angela Hilbert, Tilman Bauer, Markus Antwerpen, Hermann Meyer, Mathias C. Walter, Johannes K.-M. Knobloch

**Affiliations:** 1 Bundeswehr Institute of Microbiology, Munich, Germany; 2 TIB MOLBIOL GmbH, Berlin, Germany; 3 Friedrich-Loeffler-Institute, Federal Research Institute for Animal Health, Wusterhausen, Germany; 4 Freiburg, Germany; 5 Institute of Bioinformatics and Systems Biology, Helmholtz Zentrum München, German Research Center for Environmental Health, Neuherberg, Germany; 6 Department of Genome-Oriented Bioinformatics, Center of Life and Food Science Weihenstephan, Technische Universität München, Freising, Germany; 7 University of Lübeck, Institute for Medical Microbiology and Hygiene, Lübeck, Germany; University of Minnesota, United States of America

## Abstract

The acute disease antigen A (*adaA*) gene is believed to be associated with *Coxiella burnetii* strains causing acute Q fever. The detailed analysis of the *adaA* genomic region of 23 human- and 86 animal-derived *C. burnetii* isolates presented in this study reveals a much more polymorphic appearance and distribution of the *adaA* gene, resulting in a classification of *C. burnetii* strains of better differentiation than previously anticipated. Three different genomic variants of the *adaA* gene were identified which could be detected in isolates from acute and chronic patients, rendering the association of *adaA* positive strains with acute Q fever disease disputable. In addition, all *adaA* positive strains in humans and animals showed the occurrence of the QpH1 plasmid. All *adaA* positive isolates of acute human patients except one showed a distinct SNP variation at position 431, also predominant in sheep strains, which correlates well with the observation that sheep are a major source of human infection. Furthermore, the phylogenetic analysis of the *adaA* gene revealed three deletion events and supported the hypothesis that strain Dugway 5J108-111 might be the ancestor of all known *C. burnetii* strains. Based on our findings, we could confirm the QpDV group and we were able to define a new genotypic cluster. The *adaA* gene polymorphisms shown here improve molecular typing of Q fever, and give new insights into microevolutionary adaption processes in *C. burnetii*.

## Introduction

*Coxiella (C.) burnetii* is an obligate intracellular bacterium causing Q fever (query fever), a zoonotic disease which is ubiquitous throughout the world with exception of New Zealand. Primary reservoirs of *C. burnetii* are cattle, goats, sheep, and ticks. Other species including fish, birds, rodents, cats, and even arthropods are known to become infected. Predominantly in small ruminants, infection of adult animals is asymptomatic. However, it can lead to abortion in female ruminants and dispersion of large concentrations of *C. burnetii* by amniotic fluids and the placenta. The bacteria may also be excreted in milk, urine, and feces of infected animals. The main source of human infections is contact with infected sheep during lambing or the inhalation of dried tick feces [1], [2].

Human infections with *C. burnetii* are usually self-limiting and are associated with fever, fatigue, headache, as well as myalgia. In cases of acute Q fever, atypical pneumonia and hepatitis have been reported. Infections persisting for more than six months are regarded as chronic Q fever. The clinical symptoms are of more severe nature including endocarditis, chronic hepatitis, osteomyelitis, and septic arthritis, infection of aneurysm or vascular grafts [3]–[5]. Risk factors for the development of chronic Q fever are underlying vascular or cardiac disease, immunosuppression or pregnancy. During pregnancy, Q fever may cause premature birth, abortion, or neonatal death [6].

Several studies have tried to identify molecular markers related to the different clinical manifestations of acute and chronic Q fever [7]. One such approach was a classification based on the presence of different plasmid DNA [8]. Of the four plasmids QpH1, QpRS, QpDG and QpDV [8]–[11] and the plasmid DNA-derived sequence integrated into the chromosome (“plasmidless” strains [12]), only QpH1 was reported to be associated with acute and QpRS with chronic Q fever [8], [10]. However, this correlation was not confirmed by other researchers. Instead, host-dependent risk factors were determined to be responsible for an acute or chronic outcome of infection with *C. burnetii* [13], [14]. Furthermore, plasmid-related classification was found to correlate well with the genomic groups I to VI postulated after RFLP- and microarray based experiments [15], [16]. QpH1 forms the group I-III, QpRS represents group IV, the plasmidless isolates could be assigned to group V, and the special Dugway group, with the QpDG plasmid, forms cluster VI. Until now, the QpDV plasmid could not be associated with a distinct genomic group.

In 1998, To and colleagues [17] identified a 28 kDa protein (P28) that was immunodominant in isolates from patients with acute Q fever. The same group identified the gene and proposed to use the chromosomal acute disease antigen A (*adaA*) as a diagnostic marker for acute Q fever [18]. The gene has an open reading frame (ORF) consisting of 684 bp, coding for the hypothetical protein CBU_0952 (NP_819961), which was identified in a recent study [19].

The experimental basis for the correlation between *adaA* and the progress of disease were ten human isolates only: four of them from acute and six originating from chronic cases, as well as 11 from animals [18], [20]. In this study we investigated 23 strains from human patients and 86 strains of animal origin. We also included nucleotide sequences of seven published genomes [19], [21], [22] resulting in an improved classification of the *adaA* region. Phylogenetic analysis showed a good correlation to published whole genome data comparison including genomic grouping providing new information on microevolutionary events.

## Materials and Methods

### Strains and DNA Extraction

All but one *C. burnetii* isolates from different hosts and geographical regions (Table 1 and 2) were propagated in Buffalo Green Monkey (BGM) cell cultures under biosafety level 3 (BSL-3) conditions. Heat-inactivation was performed as described elsewhere [14], [23]. All strains were identified as *C. burnetii* by amplifying regions of the insertion sequence IS1111a [19], [24] in a real-time PCR assay as described previously [25]. DNA was extracted with the MagNA-Pure-Compact-System (Roche Diagnostics, Mannheim, Germany) according to the manufacturer's instructions. *C burnetii* DNA from a pregnant woman with chronic Q fever was extracted from paraffin-embedded placental tissue, as described previously [26].

**Table 1:** *adaA* genotypes of human *Coxiella burnetii* isolates from acute and chronic Q fever.

Strains	Source/Origin	Tissue	Course/Disease	Reference	Plasmid	*adaA* genotype^c^	GenBank accession
CS-L35	Slovakia, 1954		acute	[23]	QpH1	*adaA*	JQ713158
CS-Florian	Slovakia, 1956	blood	acute	[23]	QpH1	*adaA*_SNP_	JQ713159
Henzerling	Italy, 1945	blood	acute	[23]	QpH1	*adaA*_SNP_	JQ713160
Herzberg	Greece		acute	[23]	QpH1	*adaA*_SNP_	JQ713161
Balaceanu	Romania		pneumonia	[23]	QpH1	*adaA*_SNP_	JQ713162
Brasov	Romania		pneumonia	[23]	QpH1	*adaA*_SNP_	JQ713163
Geier	Romania		pneumonia	[23]	QpH1	*adaA*_SNP_	JQ713164
Stanica	Romania		pneumonia	[23]	QpH1	*adaA*_SNP_	JQ713165
Utvinis	Romania		pneumonia	[23]	QpH1	*adaA*_SNP_	JQ713166
290/03	Germany, 2003	lung	pneumonia	this study	QpH1	*adaA*_SNP_	JQ713167
F2	France, 1991	blood	acute hepatitis	[14]	QpDV	Q154-del, B_1_	JQ713155
F9	France, 1992	blood	acute hepatitis	[14]	QpDV	Q154-del, B_1_	JQ713149
RT-Schperling	Kyrgyzstan, 1955	blood	acute	[23]	QpDV	Q154-del, B_1_	JQ713150
RT 1140	Crimea, Ukraine, 1954	blood	pneumonia	[23]	QpDV	Q154-del, B_1_	JQ713145
F1	France, 1992	aortic valve	endocarditis	[14]	QpRS	Q154-del, B_1_	JQ713152
F3	France, 1991	mitral valve	endocarditis	[14]	QpRS	Q154-del, A	JQ713154
F7	France, 1992	mitral valve	endocarditis	[14]	QpRS	Q154-del, A	JQ713144
Z416/96	Saudi-Arabia, 1996	blood	endocarditis	[39]	QpRS	Q154-del, A	JQ713143
F4	France, 1992	blood	endocarditis	[14]	QpDV	Q154-del, B_1_	JQ713153
Scurry Q217	USA, 1981	liver	chronic hepatitis	[8]	plasmidless^b^	Q212-del	JQ713156
F5	France, 1991	blood	endocarditis	[14]	QpH1	*adaA*	JQ713168
F10	France, 1992	aortic valve	endocarditis	[14]	QpH1	*adaA*_rep_	JQ713169
99/3^a^	Germany, 1999	placenta	endocarditis, abortion	this study	QpH1	*adaA*_SNP_	JQ713170

Chronic isolates are listed from entry F1 onwards.

a DNA was isolated from paraffin-embedded material.

b no plasmid detected, but plasmid-related sequences present in genomic DNA.

c *adaA*: intact *adaA* gene (reference RSA493); *adaA*_SNP_: *adaA* gene with A/T SNP at position 431; *adaA*_rep_: *adaA* with 226 bp tandem repeat; Q154-del: Q154-type deletion of *adaA*; Q212-del: Q212-deletion type of *adaA.*

**Table 2:** *adaA* genotypes in 86 animal-derived *Coxiella burnetii* strains.

Number of strains	Species	Disease/source	Plasmid type^a^	*adaA* genotype^b^
26	Cow	abortion, milk	QpH1	*adaA*
6	Cow	abortion, milk	QpH1	*adaA*_SNP_
1	Sheep	abortion	QpH1	*adaA*
18	Sheep	abortion	QpH1	*adaA*_SNP_
1	Sheep	abortion	QpH1	*adaA*_rep_
1	Sheep	abortion	QpRS	Q154-del
4	Goat	abortion	QpH1	*adaA*_SNP_
2	Goat	abortion	QpH1	*adaA*_rep_
2	Goat	abortion	QpRS	Q154-del
13	Tick	–	QpH1	*adaA*
8	Tick	–	QpH1	*adaA*_SNP_
1	Tick	–	QpH1	Q154-del
1	Tick	–	QpH1	Q212-del
1	Mouse	spleen	QpH1	*adaA*_SNP_
1	Fallow deer	abortion	QpH1	*adaA*_SNP_

a the plasmid type had been described elsewhere [6], [9], [40].

b *adaA*: intact *adaA* gene; *adaA*_SNP_: *adaA* gene with A/T SNP at position 431; *adaA*_rep_: *adaA* with 226 bp tandem repeat; Q154-del: Q154-type deletion of *adaA*; Q212-del: Q212-deletion type of *adaA* (see also Figure 1). The GenBank accessions for the Q154-type deletion are JQ713146 (Z 3574), JQ713148 (Namibia), AAUP02000002@complement (10798.12509) (Priscilla Q177), and JQ713151 (Z 5a). The accession for the Q212-type deletion is JQ713157 (Z 11).

Fifty different bacterial species used as negative controls were obtained from the German national strain collection (DSMZ, Braunschweig, Germany) or from different clinical sources [27].

### PCR Assays

To generate a reliable tool for the detection and identification of the *adaA* gene, a real-time PCR assay with HybProbe-technology (Roche) was designed. For this, the *adaA* ORF (684 bp) was cloned from the reference strain (Nine Mile RSA493; NC_002971) into *E. coli* and confirmed by DNA sequencing (GenExpress, Berlin, Germany). Seven different primers and three different sets of hybridization probes were selected and tested with the cloned target as template (data not shown). The best results were obtained with primers *adaA*_S and *adaA*_R, which generated a fragment of 127 bp in length (NC_002971 position: 902656-782), using 0.5 µM of primers and 0.15 µM of probes *adaA*_FL and *adaA*_LC with FastStart DNA Master PLUS (Roche, Mannheim, Germany) in a volume of 20 µl. After 10 min at 95°C for polymerase activation, 45 amplification cycles were performed, each with 10 s denaturation at 95°C, 8 s annealing at 55°C and 15 s elongation at 72°C on a LightCycler 1.2 instrument (Roche, detection in channel F2). Melting curve analysis comprised of 20 s denaturation at 95°C and 20 s incubation at 40°C by continuous heating to 85°C with a slope of 0.2°C/s was performed afterwards.

The plasmids QpH1 and QpRS were determined by conventional PCR as described elsewhere [28]. Two new PCR assays were designed to identify the QpDV plasmid and the “plasmidless” type (Table 3). The PCR conditions of these assays are as follows: After 10 min at 95°C for FastStart Taq DNA polymerase activation, 45 amplification cycles were performed, each with 10 s denaturation at 95°C, 10 s annealing at 55°C and 11 s elongation at 72°C on a LightCycler 1.2 instrument. Again, a melting curve analysis was completed after 20 s denaturation at 95°C and 30 s incubation at 40°C by continuous heating to 95°C with a slope of 0.2°C/s.

**Table 3:** Primers and probes used in this study.

Primer	Sequence (5′–3′)	Amplicon size (bp)
**amplification**		
*adaA*_R	TTCTTTTTggTTAgCggCgTAg	127
*adaA*_S	CCAgCgAgTTTACgATCAAg	
*adaA*_LC	LC640-AgCATCgATTTTgTCTCTCCACCg-PH	53
*adaA*_FL	CgTTTCCgAATggTCATTATTTTTTAC-FL	
L4	TggCATATTTgTTACTTgCg	7462
R4	ACTgCATCgTgAggTTgCAg	
L4 nested	AAgAATTTgTAgCAgAAATA	7429
R4 nested	gAggTTgCAgAAgAAgTggg	
Cox *adaA*_*	AACTTTTCTAgCgTTATTTgCCTAT	718
Cox *adaA*_ATG	AggAggAggTCACTTgAAAAAACTA	
**sequencing**		
M13-FP	TgTAAAACgACggCCAgT	
M13-RP	CAggAAACAgCTATgACC	
P_a3_	TTCTTCATCggTgCTATg	671
P_a4_	ATCgTTATCTgCATCCTgC	
**QpDV**		
QpDV_F	CTTATTTCAAAgAgTTCCTgCTAg	166
QpDV_R	CgCAACCggCTgTTgTgC	
QpDV_FL	TACgTATgAACCgCAgAATACCg-FL	53
QpDV_LC	LC Red640-TCCCTTggAAAggAATgCTAgAAATTg-PH	
**plasmidless** a		
Integr_S	AgCgATAAATgAAgTAATgCCgT	214
Integr_R	ATATTCTgTATTAATCgAAAgCgAg	
Integr_FL	TTTTTATTgATCgCCAATTAgTATggT-FL	54
Integr_LC	LC Red640-CTTgTTgAACATCAATCACgTCgTT-PH	

a no plasmid detected, but plasmid-related sequences present in genomic DNA.

For analyzing the region flanking the *adaA* gene deletion, an extended PCR-primer pair system comprising primer R4 and L4 was designed. The PCR was performed on a GeneAmp PCR-system 9700 (PE Applied Biosystems, USA) with the Expand Long Template Mix, buffer 2 (Roche, Mannheim, Germany) and Clontech Polymerase BD-Advantage (Becton-Dickinson, Heidelberg, Germany). The cycling protocol encompassed 150 s at 95°C, 35 cycles with 30 s at 95°C, 30 s at 53°C, 8 min at 68°C and a final extension for 7 min at 68°C. Chromosomal DNA of Nine Mile wild type was used as positive control for all PCR reactions (amplicon size of approx. 8 kb). For further amplification of the specific PCR product, a nested PCR was performed with the primers L4 nested and R4 nested using the same instruments and chemistry.

### Determination of Sequence Polymorphisms

After identification of *adaA* positive strains, we performed a second PCR using the primers Cox *adaA*_* and Cox *adaA*_ATG with BigDye Terminator chemistry (Applied Biosystems/Life Technologies, Darmstadt, Germany) on a 3100 ABI sequencer (Applied Biosystems/Life Technologies, Darmstadt, Germany) in order to identify sequence variations in the entire *adaA* gene. To analyze the *adaA* deletion regions, PCR-products generated with primers L4 nested and R4 nested were cloned into Plasmid pAlli10 according to the kit manual (Alligator cloning kit; Trenzyme, Constance, Germany). Sequencing reactions in an ABI 3300 sequencer (Applied Biosystem, Darmstadt, Germany) were performed with primers M13-FP, M13-RP, P_a3_, and P_a4_ to cover the entire region of the deletion mutants. Sequence analysis was performed using the Software packages of Vector NTI (version 9.0; Invitrogen, Carlsbad, CA, USA) and MEGA (version 5.03, Center for Evolutionary Medicine and Informatics, Tempe, USA [http://www.megasoftware.net]).

### Phylogenetic Analysis

An *in silico* analysis of the seven reference genomes was performed using the genome aligner Mauve [29]. Based on the computed multiple sequence alignment of the *adaA* chromosomal region, long collinear blocks (LCBs) were used to identify syntenic regions across two or more genomes (see Figure 1). The comparison of deletion types presented in strains from our collection, shown in Figure 2, was performed on an end-trimmed multiple sequence alignment computed with MAFFT [30]. Phylogenetic trees were calculated based on the multiple sequence alignments using the maximum parsimony criterion as implemented in PAUP* [31]. Thereby, deletions were encoded as additional states according to [32] using SeqState (version 1.4.1, [33]). To root the trees, Dugway was set as outgroup. Bootstrap values were computed based on 1,000 samples and were higher than 90% for all branches.

**Figure 1:**
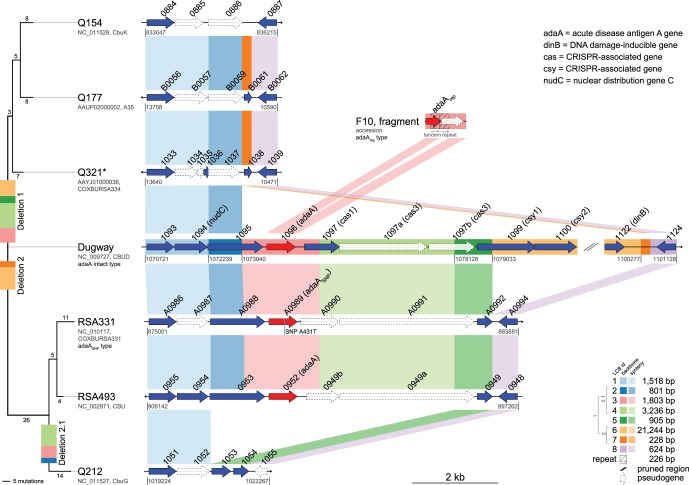
Polymorphisms of the *adaA* genetic region demonstrated using seven *C. burnetii* reference genomes. Three deletion events resulting in two main deletion types (Q154 and Q212) and three *adaA* gene variants (*adaA*, *adaA*_SNP_ and *adaA*_rep_) resulting from independent mutation processes were identified *in silico*. The organization of the *adaA* flanking region of the Dugway genome (displayed in the center) is compared to the other six reference genomes. Just for the purpose of visual compactness, the light orange region was pruned between the ORFs CBUD_1100 and CBUD_1122. Gene annotations are obtained from GenBank. Coding genes are drawn in blue, pseudogenes in white and the *adaA* gene in red. Arrow direction represents the location of the ORFs at the forward and backward strand. Locus tags are shortened by its common prefix and written above the ORFs. If gene names are known, they are written next to their locus tag and in parentheses. Long collinear blocks (LCBs) are shown as colored rectangles at the backbone of the Dugway genome. The pairwise syntenic regions between the genomes are drawn in lighter color, accordingly. The phylogenetic relationship is shown at the left side. Just for visualization purposes, the Dugway genome was rotated to the middle. The length of the branches encodes the number of mutation events. Three different deletions were identified and its affected LCBs are drawn to the phylogenetic branch where the deletion events may have occurred. Dugway and RSA331 were chosen as representative strains to show the intact *adaA* gene and *adaA*_SNP_ gene, respectively. A fragment of strain F10 is exposed in the figure to show the inserted repeat variant (*adaA*_rep_). The figure was drawn using genoPlotR [37]. *Q321 replaces RSA334, which was accidently assigned to the published whole genome data.

**Figure 2:**
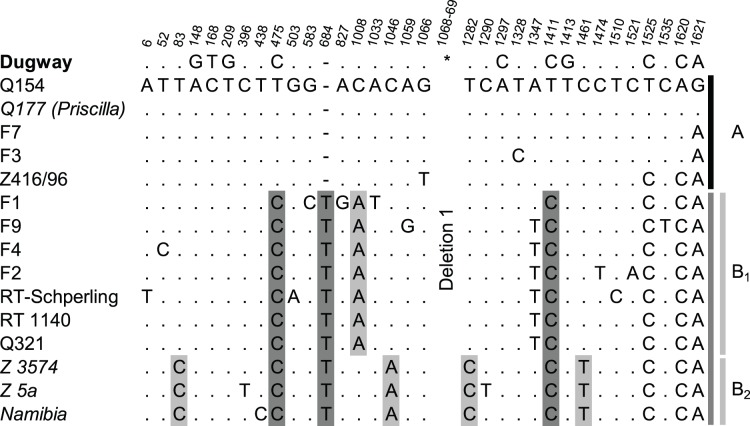
Sequence-based discrimination of the Q154 *adaA* gene deletion type. This special deletion type is defined by deletion 1 (see Figure 1). Differential bases (SNPs and insertion) of analyzed strains are shown in reference to the published genome of Q154, position 834,296 to 836,007. Vertical numbers display the respective position relative to position in the genome of strain Q154. The large deletion 1 is indicated with an asterix (*). Three conserved distinctions defining the subgroup B are marked in dark grey. The insertion of a ‘T’ is located between position 834,978 and 834,979 of the Q154 genome. Subgroup B can be further divided into two groups B_1_ and B_2_ (light grey lines) by 5 conserved SNPs (marked light grey).

## Results

### Specificity and Reproducibility of the Real Time PCR Assay

The *adaA*_S and *adaA*_R primers and probes were specific for all 94 *adaA* positive *C. burnetii* strains investigated. The *C. burnetii* reference strain (Nine Mile RSA493) was *adaA* positive whereas the well-known strain Priscilla Q177 containing the QpRS plasmid displayed no PCR signal. DNAs from 50 other bacterial species were negative, indicating that the assay is specific (data not shown). The limit of detection was determined by a serial dilution of photometrically quantified plasmid and genomic DNA and was found to be one plasmid copy per reaction. Melting curve analysis of *C. burnetii*-derived PCR products revealed a single distinct melting peak at approximately 61°C for all investigated strains. The size of the amplicons was confirmed by gel electrophoresis and determined as 127 bp (data not shown) with the exception of 4 strains, which showed a larger product.

### Distribution of the *adaA* Gene in *C. burnetii* Isolates

Ten out of the fourteen strains obtained from human patients with acute disease were positive in the *adaA* gene real-time PCR and all of them carried the plasmid QpH1. Four strains related to fever (n = 1), pneumonia (n = 1) and hepatitis (n = 2), were negative in the *adaA* PCR assay. In contrast to the other isolates, these strains showed the QpDV plasmid type.

Six isolates from chronic cases (one chronic hepatitis and five endocarditis patients) were negative for the *adaA* gene. The plasmid types were QpRS, QpDV or “plasmidless” (strain Scurry Q217). However, two *C. burnetii* isolates from French patients with endocarditis and one abortion-derived isolate from a chronically infected patient with endocarditis and valve replacement were *adaA* positive. All of these three strains harbored the QpH1 plasmid as well (Table 1).

Eighty-one out of the 86 investigated animal strains were *adaA* positive and carried plasmid type QpH1. Three of the five negative strains isolated from goat or sheep showed the QpRS plasmid type, while the two other strains derived from tick pools, were also QpH1 positive (Table 2).

### Sequence Variations of the *adaA* Gene

Gel electrophoresis analysis of the *adaA* PCR products for some isolates showed a larger product of about 900 bp as opposed to the expected size of 684 bp. Sequence analysis of the 900 bp amplicon revealed a 226 bp duplication (base 107 to 332), resulting in a premature stop codon after 133 amino acids. Due to this observation, in all *adaA* positive strains the whole ORF was sequenced. Only one human (chronic Q fever endocarditis) and three animal isolates (two goat, one sheep) presented this variation. Further sequence analysis revealed a novel non-synonymous single nucleotide polymorphism at position A431T, resulting in an amino acid substitution of glutamic acid (E) by valine (V) – which was found in 49 strains. This included nine out of 14 isolates from acute Q fever patients and one out of eight isolates from chronic Q fever patients (Table 1). In animals, the SNP was present in all of the studied species (see Table 2).

### *adaA* Deletion Sites

All 15 *adaA* negative strains were analyzed by sequencing the products of long-range PCR targeting flanking regions of the gene. The sequences were aligned with the chromosomal *adaA* flanking region of strain Dugway and lacking regions were designated as deletions (Figure 1). Analysis of these and comparison with published *adaA* negative genomes (Q154, NC_011528 and Q212, NC_011527) revealed two differing main deletion types (Figure 1). The *adaA* gene in the reference strain Nine Mile RSA493 is located close to a described insertion site (between CBU_0948 and CBU_0949; [21]) compared to the Dugway genome comprising more than 20 putative ORFs (CBUD_1100 to CBUD_1122; NC_009727). The more common deletion 1 resulted from a large 27.1 kb deletion of the *adaA* gene, an ORF designated CBU_0949 in strain Nine Mile, and the ORFs CBUD_1100 to CBUD_1122 (Figure 1, deletion 1). This resulted in the Q154-type of *adaA* negative strains (13 strains, Tables 1, 2).

CBU_0949 annotated in strain Nine Mile is the truncated version of ORF CBUD_1099 annotated in strain Dugway which was partially deleted by the large deletion comprising the ORFs CBUD_1100 to CBUD_1122 (deletion 2). Another deletion event is seen in the strain Q212 and is the consequence of a 5,9 kb deletion (Figure 1, deletion 2.1) in a genetic background homologous to strain Nine Mile [21] remaining 801 bp upstream of deletion 1 as well as from a truncated ORF CBU_0949. This type of deletion was only seen in one human chronic Q fever isolate with liver involvement and in one tick isolate from Germany. Only strains of the Q154-deletion type show a minor difference of 228 bp in the 3′-flanking chromosomal region of the deletion 1 and 2. Comparison of all sequenced Q154-type *adaA* deletions revealed several non-coding SNPs as well as a one base-pair insertion (Figure 2). Making use of these variations, the strains of the Q154-deletion type can be differentiated further into two subgroups (A+B) by two conserved SNPs as well as the one base-pair insertion (Figure 2, dark grey). Within subgroup B, 5 SNPs were used for further differentiation (subgroups B_1_ and B_2_, Figure 2, light grey).

### Phylogenetic Analysis

**Figure 3:**
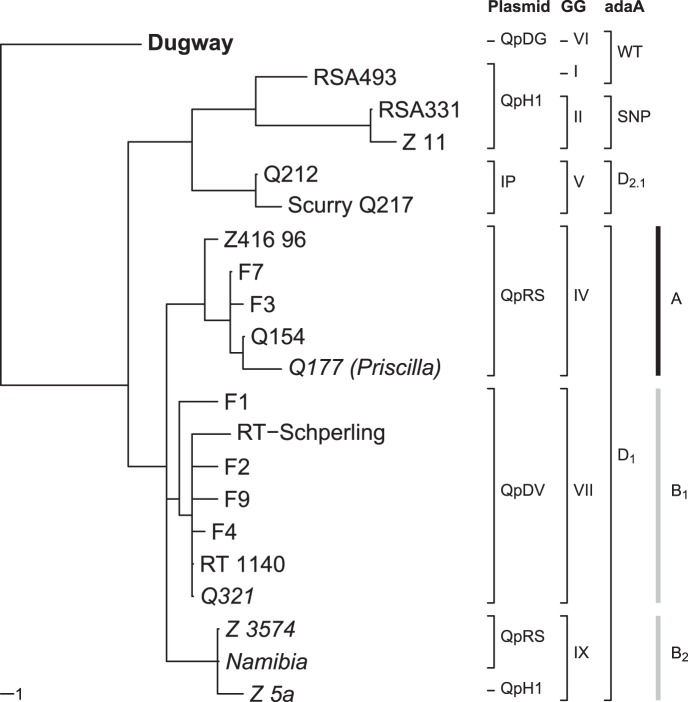
Phylogenetic relationship of studied strains. Maximum parsimony tree using 1,713 bases in the dataset computed with PAUP* and rooted using Dugway (bold) as outgroup. Plasmid type, genomic group (GG), *adaA* types and the subgroups of the Q154-deletion type are shown next to the tree. Branch lengths correspond to the number of mutations. Animal strains are written in italic. IP: integrated plasmid; WT: wildtype; D_2.1_: deletion 2.1; D_1_: deletion 1. The figure was drawn using APE [38].

The phylogenetic relationship based on *adaA* gene variants was studied using MAFFT and PAUP* and is shown in Figure 3. The analysis matches with the former RFLP-based genomic grouping I to VI [15] and the microarray-based whole-genome comparisons by Beare et al. [16]. Group I, represented by the Nine Mile/RSA493 strain is carrying the *adaA* wildtype, whilst group II represented by RSA331, carries the *adaA*_SNP_ variant. Both groups harbor the QpH1 plasmid. Group IV (QpRS associated) was formed by the Q154-deletion types and group V (“plasmidless” = Q212-deletion) and group VI (“Dugway/QpDG” = non-disrupted *adaA* region) could also be allocated to distinct *adaA* (deletion) variants. In our analysis, the genomic groups I and II are related to group V whereas IV and VI show a closer relationship to each other. In addition, another distinct group of isolates carrying the QpDV plasmid type (identical to the above described Q154_B_1_-type) also reveals a close relationship to group IV and VI as well as the newly defined Q154_B_2_ subcluster.

## Discussion

The sequence variations of the *Coxiella burnetii* specific acute disease antigen A gene, postulated to be associated with acute human Q fever [18], were completely analyzed *in silico* and *in vitro*. In contrast to previous publications, this study used a large and well-defined collection of strains from human clinical entities and different animal species to resolve the heterogeneous structure of this gene region. Using a novel real-time PCR assay, we were able to demonstrate that 10 out of 14 strains isolated from acute human Q fever patients were *adaA* positive. The *adaA* negative isolates are derived from acute Q fever patients with hepatitis (n = 2), fever (n = 1), or pneumonia (n = 1). This supports a recently published observation from a Spanish study [34], in which all strains isolated from acute Q fever hepatitis patients were *adaA* negative and only one *C. burnetii* isolate from a pneumonic patient was *adaA* positive. In our study, in only three out of nine isolates from chronic Q fever endocarditis patients, including one isolate from paraffin-embedded abortion material, the *adaA* gene was detected. Thus, our results do not support a significant association of the *adaA* gene with the acute course of Q fever.

### Plasmid Correlation

The correlation between plasmid type and *adaA* genotype present in human *C. burnetii* isolates was also observed in animal-derived strains with only very few exceptions. Sixty five out of 67 strains carrying QpH1 displayed an *adaA* positive genotype. Only two QpH1 strains, isolated from a tick pool from a region in south-western Germany, were negative. The type strain Dugway carrying the QpDG plasmid was also *adaA* positive [18]. The three strains of QpRS plasmid type were *adaA* negative, corroborating the results of the strains isolated from chronic human disease. In summary, we confirmed that in every *adaA* positive strain (except Dugway) we could detect the QpH1 plasmid as well. As no positional linkage was found between both, this cannot be sufficiently explained by molecular reasons and remains an open question.

### *adaA* Gene Variants

For the first time, our in-depth analysis showed a quite heterogeneous appearance of the structure of this genetic region. We identified five different variants: the *adaA* wildtype, a second variant with a tandem repeat, a SNP type and two different deletion types. The *adaA*-repeat type (size: 226 bp, two copies, starting at position 107) was first described by a French group in 2009 [20], and were correlated to *C. Burnetii* isolates from goats. In contrast to this, our study identified the tandem repeat also in one sheep strain and one isolate from a chronic Q fever endocarditis patient. The SNP at position 431 resulted in an amino acid substitution (glutamic acid to valine). Remarkably, this is the predominant variant in strains from acute Q fever (10 out of 11) patients, predominantly the pneumonic variant. Only one chronic sample (“99/3”) also harbored this variant, indicating that there may be an acute respiratory disease pattern before chronification. In animals, the SNP was detected in more than 50% of the analyzed isolates (49 out of 81). Regarding to the species level, the ratio is as follows: the majority of sheep isolates (18/21) carried this genotype. In goats (4/8) and cows (6/32), the presence was more varied. This correlates well with the observations, that ewes are the predominant source for Q fever outbreaks infecting humans in the past.

Using the SNP, a rough trace back analysis of Q fever was performed with the two German human isolates. The “290/03” *C. burnetii* strain was epidemiologically allocated to a major German Q fever outbreak in Soest in 2003 [35] and two *C. burnetii* isolates from a sheep flock in the same geographical region also carried the *adaA* gene SNP. In the chronic Q fever case, dedicated to the isolated DNA “99/3”, all regional sheep strains isolated prior to the beginning of the disease (before 1999) were also presenting this variant. Other possible interpretations for our findings are that humans are not as susceptible for the “normal” *adaA* gene variant as for the SNP variant, or that both variants are linked to different chromosomal changes responsible for different phenotypes in the susceptible host.

### Deletions in the *adaA* Gene Region May Reflect Phylogenetic Events in the Evolution of *C. burnetii*

We could show that the *adaA* negative status of isolates is much more complex than described to date. By comparing all of the published genomes, and the sequenced *adaA* deletion sites in this study, a clear discernible genome reduction is seen resulting in the following phylogenetic relationship placing Dugway as common ancestor (Figure 1): The 27.2 kb deletion 1 from Dugway resulted in the Q154 *adaA* deletion genotype, while the independent 21.4 kb deletion 2 in Dugway, not affecting the *adaA* gene region, resulted in Nine Mile and RSA331 (distinguishable from each other by the *adaA*_SNP_). The subsequent deletion 2.1 of 5.9 kb within Nine Mile resulted in the Q212 *adaA* deletion genotype.

The first genomic variation divides the chronic disease branch (Q154) from the acute Q fever cluster (RSA331). Further, the Q154-deletion could be subdivided by several conserved non-coding SNPs and one insertion into three other clusters (see Figure 2). Cluster A presents QpRS strains from chronic Q fever endocarditis patients, whereas the second cluster B, with multiple plasmid types, is further differentiated into human-derived isolates. The QpDV plasmid (B_1_) and an animal-based group (B_2_) harboring QpH1 and QpRS plasmids. Deletion 2.1 (5.9 kb) correlates very well with findings from a recently published paper [21] that described the lack of ORFs in strains belonging to the QpRS and other plasmid types different from QpH1. Furthermore the identified Q154- respectively Q212-deletion type in two QpH1 strains from ticks (see above) may indicate that horizontal gene transfer is possible in ticks infected by multiple *Coxiella* clones.

By looking at the few single mutation events (SNPs, InDels) in the small genomic region encoding the *adaA* gene, we could reconstruct the same phylogenetic relationship as shown previously with whole genome analysis methods [16]. This adds strongly to the evidence of the clonal evolution of *C. burnetii.*

### Genomic Grouping

Our results also correlate well with plasmid-related classification and the genomic groups I-VI postulated after RFLP and microarray based experiments [15], [16] resolving these groups further. The described distinction inside the genomic groups I to III could be confirmed for the groups I and II, where two *adaA* gene variants are seen (wildtype and SNP). In more detail, both clusters differ in 11 (RSA331) and 4 SNPs (RSA493), respectively. These groups share a hypothetical common ancestor with Q212 (group V) further divided by 14 additional SNPs and the deletion event described earlier. On the other hand, we could demonstrate a closer evolutionary relationship between group IV and VI, supporting *C. burnetii* Dugway isolate 5J108-111 as a common ancestor of all described genomic groups. Interestingly, Q321 clusters phylogenetically with a distinct group formed by QpDV-harboring strains exhibiting the *adaA* deletion type Q154_B_1_. Until today, this relevant *C. burnetii* strain cluster bearing the QpDV plasmid was not placed into a genomic group, most likely due to missing isolates in the former studies. We assume that all members of this special subdivision build the genomic group VII, formerly proposed by Beare et al. [16]. This additional evidence for the existence of the genomic group VII was confirmed by the above-mentioned Spanish study, where two isolates from our collection (F2 and F4) were placed together with a local tick cluster of 32 strains. Unfortunately, no plasmid type information was given. Hence, we can associate two of the above mentioned new Q154-deletion type subclusters with existing genomic groups IV (A) and VII (B_1_), whereas the other subcluster B_2_ can be postulated as one or two new genomic groups IX and X, depending on the occurrence of different plasmid types.

An additional analysis of the inter-species relationship of the AdaA protein with HHblits, a remote homology detection tool based on HMM-HMM [36], revealed a homologous protein in *Legionella pneumophila*, the taxonomically closest related species. Unfortunately, no function could be assigned to this protein in *Legionella* so far. Hence, the function and relevance of the AdaA protein is still unknown.

It seems that the microevolutionary process of *C. burnetii* is homogenously displaced and no clear allocation to distinct genes is possible. This could explain the observed phylogenetic relationships between strains independent of type and number of studied genes or gene regions. It fits within the thesis of a highly conserved genome in this species and is useful in molecular strain comparison studying various endemic regions and clinical entities.

Therefore, classification of *adaA* gene variants, if present in the genome, or determination of the deletion type, is an important tool for the investigation of molecular epidemiology and the evolution of Q fever.
